# Role of Alarmins in the Pathogenesis of Systemic Sclerosis

**DOI:** 10.3390/ijms21144985

**Published:** 2020-07-15

**Authors:** Antonello Giovannetti, Elisabetta Straface, Edoardo Rosato, Marco Casciaro, Giovanni Pioggia, Sebastiano Gangemi

**Affiliations:** 1 Department of Translational and Precision Medicine, Sapienza University of Rome, 00185 Rome, Italy; edoardo.rosato@uniroma1.it; 2 Center for Gender-Specific Medicine, Biomarkers Unit, Istituto Superiore di Sanità, 00161 Rome, Italy; elisabetta.straface@iss.it; 3 School and Operative Unit of Allergy and Clinical Immunology, Department of Clinical and Experimental Medicine, University of Messina, 98122 Messina, Italy; mcasciaro@unime.it (M.C.); gangemis@unime.it (S.G.); 4 Institute for Biomedical Research and Innovation (IRIB), National Research Council of Italy (CNR), 98164 Messina, Italy; giovanni.pioggia@cnr.it

**Keywords:** systemic sclerosis, alarmins, oxidative stress, fibrosis, cytokines

## Abstract

Systemic sclerosis (SSc) is a rare chronic autoimmune disease associated with significant morbidity and mortality. Two main subsets of SSc are recognized: (i) diffuse cutaneous SSc with rapidly progressive fibrosis of the skin, lungs, and other internal organs; and (ii) limited cutaneous SSc, which is dominated by vascular manifestations, with skin and organ fibrosis generally limited and slowly progressing. In spite of intense investigation, both etiology and pathogenesis of SSc are still unknown. Genetic and environmental factors, as well as abnormalities of immune functions, are strongly suggested for etiology, while microvascular abnormalities, immune system activation, and oxidative stress are suggested for the pathogenesis. Recently, it has been found that a multitude of mediators and cytokines are implicated in the fibrotic processes observed in SSc. Among these, a central role could be exerted by “alarmins”, endogenous and constitutively expressed proteins/peptides that function as an intercellular signal defense. This review describes, in a detailed manner, the role of alarmins in the pathogenesis of scleroderma.

## 1. Introduction

SSc is a rare autoimmune disease characterized by microvascular damage, fibrosis of the skin and internal organs, and aberrant immune activation. Based on the extent of skin involvement, two main subsets of SSc are recognized: (i) diffuse cutaneous SSc (dcSSc) with rapidly progressive fibrosis of the skin, lungs, and other internal organs; and (ii) limited cutaneous SSc (lcSSc), which is dominated by vascular manifestations, with skin and organ fibrosis generally limited and slowly progressing. Vascular disease is clinically revealed by digital pits and ulcers whereas visceral involvement commonly includes gastroesophageal reflux disease, interstitial lung disease, and cardiac diastolic dysfunction.

Growing evidence corroborates the notion that a complex interplay between genetic, environmental, and immunological factors plays a causative role in the development of systemic sclerosis. It is commonly accepted that SSc develops in an individual with a permissive genetic background. Thanks to the advances of genetic techniques, many SSc susceptibility loci have been firmly identified both in the MHC-HLA region and in non-HLA immune regulatory and inflammatory genes. HLA class-II is the most significant region associated with SSc, while *CD247*, encoding for the CD3 zeta chain, the interferon regulatory factor 5 (*IRF5*), and the signal transducer and activator of transcription protein 4 (*STAT4*) are the non-HLA-associated genes more frequently associated with SSc susceptibility. Taken as a whole, genetic studies have shown that virtually all SSc-associated risk loci are located in genes related to innate immune signaling, including the toll-like receptor (TLR) and interferon (IFN) systems. Variants of *TNFAIP3*, a key negative regulator of TLR signaling and its partner molecule, (TNFAIP3)-interacting protein 1 (*TNIP1*), both showed a strong association with SSc [1]. Multiple variants in *IFN* genes linked to mortality and discrete phenotypes of SSc, such as dcSSc, lcSSc, anti-DNA topoisomerase I antibody (ATA), anticentromere antibodies (ACA), and pulmonary arterial hypertension (PAH), point to the importance of the IFN pathway, both in the development and progression of SSc. Type I IFN synthesis is induced by a microbial challenge when the pattern-recognition receptors (PRRs) in the cytosol or within endosomes sense microorganisms such as bacteria, viruses, and fungi. An interferon signature is observed in most patients with SSc, as well as in patients with HIV infection, thus reinforcing the long-held hypothesis that infections might be the first trigger of SSc in genetically susceptible individuals.

The precise etiology and molecular pathogenesis of SSc are still unclear, although considerable evidence suggests that innate immune system responses are pivotal in disease initiation. Inappropriate activation of innate immune cells via PRRs, such as TLRs, leads to signaling cascades that are ultimately detrimental to the host. TLRs are germ-line-encoded PRRs that recognize components of pathogens, as well as endogenous danger signals, therefore contributing to the “sterile inflammation” [2]. They have been linked to different autoimmune diseases, including rheumatoid arthritis (RA), systemic lupus erythematosus (SLE), and SSc. TLR2, 3, 4, 7, 8, and 9 have particular relevance to SSc pathogenesis. A functional polymorphism in TLR2 associates with ATA positivity, while TLR4, which recognizes bacterial lipopolysaccharide (LPS) and a variety of endogenous ligands, synergizes with transforming grow factor β (TGF-β) to increase collagen production [3]. Other PRRs, named NLRs (NOD (nucleotide-binding and oligomerization domain)-like receptors) localize in the cytoplasm and recognize intracellular motifs. This family of receptors is composed in humans of 22 cytoplasmic proteins that, upon ligation, trigger NF-kB and mitogen-activated protein kinase (MAPK), thereby resulting in the expression of pro-inflammatory cytokines. In SSc patients, polymorphisms in NOD-, LRR- and pyrin domain-containing protein 1 (NLRP1) are associated with pulmonary fibrosis and anti-topoisomerase-positivity [4], whereas elevated levels of NOD2 and NLRP3 are observed in dermal fibroblast [5].

Vascular abnormalities are the earliest manifestations of SSc clinically presented as nailfold capillary abnormalities and Raynaud’s phenomenon. The causes of the initial vascular damage in SSc are unclear, but infectious agents, cytotoxic T cells, nitric oxide (NO)-related free radicals, and autoantibodies against endothelial cells have all been implicated [6]. The histological features of SSc vasculopathy include the decrease in the number of small vessels, dilation of capillaries, and stenosis of arterioles and small arteries. Blood vessels of SSc patients are characterized by a subendothelial accumulation of activated fibroblasts or myofibroblasts with excessive production of type I collagen (CI) and extracellular matrix (ECM) [7]. Another abnormality observed in SSc patients is the transition of endothelial cells to a mesenchymal phenotype expressing α smooth muscle actin (αSMA), vimentin, and CI. This phenomenon is probably under the control of TGF-β, but its role in SSc vasculopathy, if any, remain to be elucidated. Overall, key vascular abnormalities are intimal proliferation in the absence of significant inflammation, endothelial cell damage, defective angiogenesis, impaired vascular tone and permeability, the platelet activation, and finally the enhanced coagulation with decreased fibrinolysis. An impaired balance of vasoconstrictors (e.g., endothelin (ET) and vasodilator substances (e.g., NO) factors also participate in vascular dysfunction.

Nearly 30 years ago, Murrel associated, for the first time, the pathogenesis of SSc to oxidative stress [8]. Later, many reports have supported this hypothesis, showing remarkable evidence of oxidative stress, such as abnormalities of NO, nitric oxide synthase, and 8-isoprostane [9,10] for both lcSSc and dcSSc (for a comprehensive review see Vona et al. [11]). Reactive oxygen species (ROS) and reactive nitrogen species (RNS) are considered the background pathology involved in the development of SSc [12]. ROS are the reduced metabolites of molecular oxygen, including superoxide anion radical (O2•), hydroxyl anion (•OH), and hydrogen peroxide (H2O2). RNS are the overproduction of NO, nitrogen dioxide (NO2), and peroxynitrite (ONOO^−^), which is formed from the interaction between NO and superoxide. Both ROS and RNS can induce the production of pro-inflammatory and pro-fibrotic cytokines, such as platelet-derived growth factor (PDGF) and TGF-β; stimulate the proliferation and the activation of fibroblasts; augment the synthesis of CI; and cause vascular dysfunction. In conditions of intense production of ROS and RNS, red blood cells (RBCs) may also undergo oxidative damage and act as pro-oxidant “weapons” capable of transforming the behavior and fate of endothelial cells [13]. In SSc patients, alteration of RBCs induced by oxidative imbalance includes cytoskeleton oxidative denaturation and derangement, as well as loss of lipid asymmetry. These changes can ultimately result in the modification of RBC adhesive properties, aggregability, and deformability, all related to disease severity [14].

Aberrant immune activation, on the one hand, and vascular damage, on the other hand, lead to the third cardinal process in the pathogenesis of SSc, fibrosis. Fibrosis is the abnormal expression of collagens and other extracellular matrix proteins within the tissues, ultimately resulting in the failure of the organ or tissue. When tissues are damaged by excessive deposition of ECM, several signaling events occur in the cellular microenvironment where additional cells are recruited, attempting to “repair” the damaged tissue. This paradoxically elicits further exacerbation of the damage with the addition of more collagen and extracellular matrix. However, tissue fibrosis can also occur in the absence of any overt damage, and indeed, in many instances, the cause of the fibrosis remains unknown. In the tissue, effector cells responsible for fibrosis include bone-marrow-derived mesenchymal progenitors, such as fibrocytes and monocytes, and most importantly, myofibroblasts. In SSc, fibrosis occurs prevailingly in the skin and lungs, but it can also affect other tissues, such as the myocardium, gastrointestinal tract, renal interstitium, tendons, and muscles, thus contributing to morbidity and mortality [15]. A large number of mediators and cytokines and their downstream signaling cascades are implicated in the fibrotic processes observed in scleroderma. Innate immune responses have recently emerged as pivotal drivers of persistent fibrotic response in SSc. The expression of TLR4, as well as several endogenous danger signals ligand, is elevated in lesional tissue from patients with SSc, and its activation triggers fibrotic gene expression and myofibroblast transformation and survival. These endogenous danger signals are called alarmins, and together with exogenous pathogen-associated molecular patterns (PAMPs), which are microbial in origin, are referred to as damage-associated molecular patterns (DAMPs) [16,17]. Nevertheless, the terms “alarmins” and “DAMPS” are often used as synonyms.

Alarmins are endogenous and constitutively expressed proteins/peptides showing immune-activating activities. They are released in the microenvironment as a result of degranulation, cell injury, or death, or in response to immune induction [18,19]. In addition to a physiological, often homeostatic, role inside the cell, alarmins also deliver, when exposed to the extracellular milieu, danger signals to the host, triggering a local inflammatory response, as well as innate/adaptive immune responses. Most alarmins are passively released from dead cells, but some alarmins can also be actively secreted to signal early a state of sublethal cell stress [20]. Sterile tissue injury leads to the generation of DAMPs that enable cells to sense and respond to danger. However, as a consequence of severe injuries or maximal stimulation, the signaling pathways activated by alarmins may become dysregulated, resulting in unwanted pathologic inflammation. Since DAMP-initiated inflammatory responses are independent of pathogen infection, they are referred to as sterile inflammation [21]. Alarmins function as intercellular signals’ defense by interacting with chemotactic and PRRs. Based on their localization, PRRs may be divided into (i) membrane-bound PRRs, such as TLR and C-type lectin receptors (CLRs), and (ii) cytoplasmic PRRs, such as NLRs and RIG-I-like receptors (RLRs). In addition, alarmins can be sensed by several other receptors, referred to as non-PRR DAMP receptors. These include receptors for advanced glycation end products (RAGE), triggering receptors expressed on myeloid cells (TREMs), and several G-protein-coupled receptors (GPCRs). PRRs also bind PAMPs, exogenous warning signals that alert the organism to intruding pathogens, represented by microbial molecules that share recognizable biochemical features [22]. The main differences between alarmins and PAMPs are the diverse localization (endogenous vs. exogenous) and the different way of action (cytokine-like vs. receptorial) [19]. Similar to the pathogen-induced inflammation, alarmins can activate both innate immune cells, such as neutrophils, macrophages, and dendritic cells (DCs), and non-immune cells, such as epithelial cells, endothelial cells, and fibroblasts. This activation leads to the production of several cytokines and chemokines, which in turn recruit inflammatory cells and activate adaptive immune responses. Sterile inflammation is essential for tissue repair and regeneration, but when it becomes uncontrolled, sterile inflammatory diseases may arise, including metabolic disorders, neurodegenerative diseases, autoimmune diseases, and cancer [23]. A strategy evolved by DAMP-sensing receptors is to sense various DAMPs to initiate sterile inflammatory responses. In addition, the same DAMP is recognized by two or more DAMP-sensing receptors that cooperate to activate and maintain multiple effector responses (e.g., high-mobility group box-1 (HGMB-1) that binds to TLR2, TLR4, RAGE, and TREM1). Moreover, in order to amplify their responses, different DAMPs can interact with each other, (e.g., HMGB-1 bind to endogenous DNA and augment DNA-induced TLR9 activation and cytokine release) [24].

In addition to the initially described roles in host defense and cellular homeostasis, alarmins play key roles in a very broad range of physiological and pathological processes, such as gene expression, wound healing, inflammation, allergy, autoimmunity, and oncogenesis. Alarmins activate tissue-resident leukocytes, stimulating the production of a variety of inflammatory cytokines (for a comprehensive review, see [19]) and participate in the activation of inflammasomes, specialized proteins containing NLRs [25], a critical step for innate/inflammatory responses and host defense. However, inflammasomes are not only sensors for pathogens but can also be activated, as in the case of the NLRP3 inflammasome, by generic stress signals such as perturbations in reactive oxygen species and potassium concentration [26]. A critical step for the activation of the inflammasome is the cleavage and activation of caspase-1 [27]. Once activated, the inflammasome can process a large array of precursors, many of them involved in wound healing [28] and fibrosis. Many studies point out a pivotal role for the NLRP3 inflammasome in the signaling process leading to fibrosis. Considering that oxidative stress has a key role in fibrosis, it has been hypothesized that an increase of NLRP3 activation in fibrotic diseases might be due to ROS production. Many alarmins display intrinsic chemotactic activity toward different types of leukocytes, thereby contributing to the cellular infiltration into sites of infection or tissue injury. However, alarmins may also promote the recruitment of leukocytes indirectly, through the production of chemokines by activated leukocytes or upregulating the expression of adhesion molecules in endothelial cells and leukocytes, as in the case of HMGB-1, which stimulates endothelial expression of intercellular adhesion molecule 1 (ICAM-1), and β1 and β2 integrins [29].

**Figure 1:**
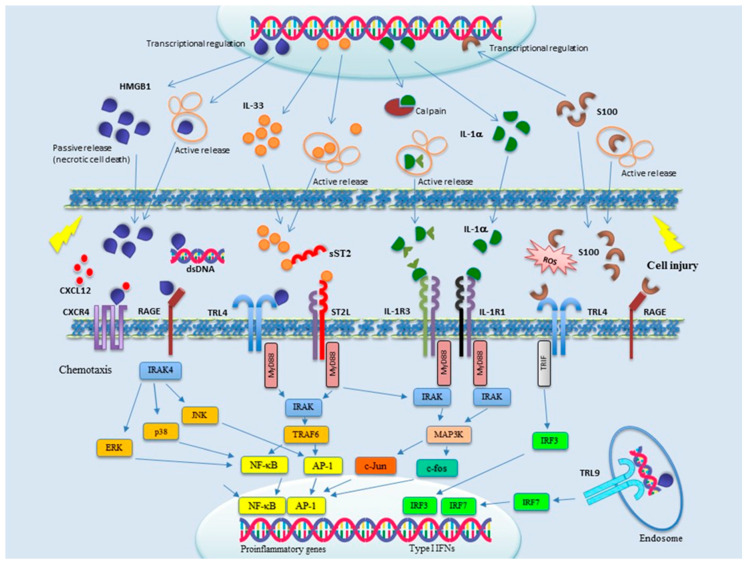
Signal transduction of the main alarmins involved in SSc pathogenesis. Upon cell injury, alarmins are released both actively and passively into the microenvironment, where they are sensed by membrane-bound receptors mainly belonging to the toll-like receptor (TLR) and IL-1R families. Multiple signal transduction pathways are thereby activated, culminating in the transcriptional activation of proinflammatory and type I interferons (IFNs) genes. Alarmins are therefore attractive targets for novel pharmacological intervention, but only a few clinical trials are currently ongoing to evaluate the effects of alarmin blockade on human diseases.

Alarmins can be grouped in three main categories: (1) nuclear, including HMGB-1, HMGN1, IL-33, and IL-1α; (2) granule derived, including α- and β-defensins, cathelicidin (LL37/cathelicidin-related antimicrobial peptide (CRAMP), eosinophil-derived neurotoxin (EDN), and granulisin; and (3) cytoplasmic, such as heat-shock protein (HSP-60, -70, -90, and -96), S100 proteins, ATP, and uric acid. Accumulating evidence indicates alarmins as primary players in SSc, as well as in many other diseases linked to inflammation and immune system activation [30]. In particular, a role for alarmins has been strongly suggested in SSc for the pathogenesis of vasculopathy, inflammation, and fibrosis. During the last few years, inhibitors of alarmin signaling have also been identified, making them attractive therapeutic tools. Signal transduction pathways of the main alarmins involved in SSc pathogenesis are schematically shown in Figure 1. In this review, we describe current knowledge about the different alarmins and the pathogenesis of SSc. New therapeutic strategies aimed to counteract alarmin functions are also summarized.

## 2. The Expression and Function of Nuclear Alarmins HMGB-1, IL-33, and IL-1α and Their Receptors in SSc

HMGB-1 is a highly conserved, non-histone, ubiquitous nuclear DNA-binding protein contained in most cell types, and it serves as a nuclear/transcriptional regulator [31]. It has both chemoattractant and activating effects on leukocytes, including dendritic cells (DCs), and hence, it has the capacity to induce innate and adaptive immune responses [19]. As a result of cell damage or via regulated secretion, similar to other “multitasking” alarmins, HMGB-1 is released into the extracellular microenvironment [32], where it acquires cytokine-like and pro-inflammatory activities [33,34,35,36,37,38,39,40]. HMGB-1 contains three residues of cysteine that determine the binding specificity to three different receptors: TLR4/MD-2 receptor axis, leading to the induction of inflammatory cytokines in macrophages [33,35]; CXCR4 to promote chemotactic cell migration of monocytes/macrophages and T cells [41]; and receptor of advanced glycation end products (RAGE) to trigger cell migration or autophagy. RAGE and TLR4 are also multitaskers, since they bind other alarmins, such as the S100 proteins [42,43] and heat-shock proteins (HSPs). RAGE belongs to the immunoglobulin (Ig) superfamily and has been described as a pattern-recognition receptor [44]. It is expressed on several cell types (monocytes/macrophages, T-lymphocytes, endothelial cells, dendritic cells, fibroblasts, smooth muscle cells, neuronal cells, glia cells, chondrocytes, and keratinocytes) and recognizes a large number of different ligands (AGEs, amyloid β peptide, S100/calgranulin protein, HMGB1, and LPS) [45]. There are two types of RAGE: (i) full-length RAGE (fl-RAGE), a transmembrane protein with a short cytoplasmic domain, essential for activation of nuclear factor-κB (NF-κB) [46]; and (ii) soluble RAGE (sRAGE), produced by alternative splicing of RAGE messenger RNA [47,48]. It has been shown that sRAGE prevents ligands to interact with RAGE or with other cell surface receptors [49], suggesting the presence of a negative feedback mechanism in RAGE signaling.

Signaling pathway activated by HMGB-1 induces the NFκB phosphorylation, which in turn results in the production of several cytokines and chemokines, such as TNF-α, IL-1β, IL-6, macrophage inflammatory protein-1α, and transforming growth factor-β by a large number of cells, including endothelial cells, fibroblasts, macrophages, monocytes, T cells, and B cells [27,37,50,51,52]. It is now well established that HMGB-1 actively promotes fibrosis. This has been observed in different pathological conditions, including pulmonary, renal, and myocardial fibrosis [53,54,55]. HMGB-1 also promotes fibrogenesis after endothelial cell damage and favors neurovascular remodeling and functional recovery after stroke and brain injury [56]. Moreover, the upregulation of RAGE observed in various diseases (rheumatoid arthritis, inflammatory kidney disease, arteriosclerosis, and inflammatory bowel disease), in association with the capacity of RAGE to bind many proinflammatory ligands (amyloid-β fibrils, S100 proteins, and HMGB-1), strongly suggests that RAGE also plays a pivotal role in the activation and maintenance of immune/inflammatory responses.

Yoshizaki et al., for the first time, observed that serum HMGB-1 and sRAGE levels were higher in SSc patients than in controls. Elevated levels of both HMGB-1 and sRAGE were also observed in bleomycin-induced scleroderma mice, an animal model of SSc. SSc patients with increased HMGB-1 and sRAGE levels also showed a more severe disease compared to those with normal levels. Lungs, heart, kidneys, and joints involvement were all associated with significantly increased levels of both HMGB-1 and sRAGE. Moreover, a significant association was also observed with anti-topoisomerase I antibodies, erythrocyte sedimentation rate (ESR), and C reactive protein (CRP). Finally, HMGB-1 and sRAGE levels were positively associated with modified Rodnan total skin thickness score and inversely with pulmonary function test [57]. A role for the higher production of reactive oxygen species in the increased HMGB-1 levels was suggested, due to vascular ischemia and reperfusion injury after Raynaud’s phenomenon. The generation of sRAGE would be a counter-system versus HMGB-1/RAGE-induced inflammatory responses.

An enhanced activation of platelets and increased tendency to aggregation have long been observed in SSc patients [58,59,60,61,62]. This is generally attributed to the concomitant dysfunction of the endothelium [63]. Activated platelets release in the plasma of SSc patients several factors involved in SSc pathogenesis, such as vascular endothelial growth factor (VEGF), PDGF, TGF-β, serotonin [64], HMGB-1 protein [65], and microparticles (MP). Microparticles, in particular, are abundant and contain bioactive HMGB-1 [66,67].

Maugeri et al. [68] found that, in the blood of SSc patients, activated platelets release abundant MP that interacts with neutrophils, promoting neutrophil autophagy, a process by which stressed cells provide anabolic substrates to feed their bioenergetics and to generate neutrophil extracellular traps (NETs), a source of autoantigens that play an important role in the pathogenesis of autoimmune diseases, like systemic lupus erythematosus (SLE) and rheumatoid arthritis [69,70]. NETs and autophagic neutrophils are abundant in blood from SSc patients. When injected into NSG mice, platelet-derived microparticles obtained from SSc patients prompt neutrophil activation and NET production, resulting, in turn, in endothelial damage and fibrosis. Microparticle–neutrophil interaction, neutrophil autophagy and survival, and accumulation in the blood of NET by-product generation were all abated in the presence of BoxA, a competitive inhibitor of HMGB-1, indicating that HMGB-1 was required for the in vivo effects of microparticles. In contrast, microparticles retrieved from the plasma of healthy controls did not cause endothelial damage in mice. Therefore, HMGB-1 is ultimately responsible for the endothelial damage in SSc [71]

Vogel et al., generating transgenic mice with platelet-specific ablation of HMGB-1 and studying trauma patients, demonstrated that platelet-derived HMGB-1 is a critical mediator of thrombosis and a regulator for platelet activation, granule secretion, adhesion, and spreading. These consequences were guided via TLR4- and MyD88-dependent recruitment of platelet guanylyl cyclase (GC) toward the plasma membrane, followed by MyD88/GC complex formation and activation of the cGMP-dependent protein kinase I (cGKI) [72]. Similarly, Stark et al., identified blood-derived HMGB-1 to be the main controller of the prothrombotic cascade, implicating platelets and myeloid leukocytes fostering occlusive DVT production [73]. Therefore, it can be speculated that HMGB-1 might coordinate microthrombosis in SSc patients and contribute to sustaining the vasculopathy associated with the disease.

Many studies have found a significant relationship also between TLR-4 and SSc. In response to PAMPs, TLR4 forms a complex with its co-receptor MD2 on the cell surface. TLR4 and its co-receptors, MD2, and CD14, were elevated in lesional skin biopsies from patients with diffuse cutaneous SSc, and significantly correlated with disease progression [74]. Interestingly, in lesional biopsies, TLR4 co-localized with myofibroblasts, as well as infiltrating macrophages and vascular cells. Increased TLR4 expression was also observed in lung biopsies from SSc-ILD patients mainly in parenchymal fibroblasts and infiltrating cells located at fibrotic loci. However, according to current evidence, the role of TLR4 in lung fibrosis is contradictory. The pulmonary fibrosis was ameliorated by TLR4 knockout in murine models of scleroderma, as well as in mice with TLR4 deleted using small hairpin RNA (shRNA) [75,76].

These findings were not confirmed by subsequent studies showing worsening of bleomycin-induced lung inflammation and fibrosis in *Tlr4*−/− mice, possibly due to an impairment of alveolar epithelial cell renewal in the absence of TLR4 [77,78]. The contrasting observations in these studies are difficult to reconcile at the moment. In skin, fibroblast treatment with LPS, or endogenous TLR4 ligands, triggered a profibrotic gene expression program and transdifferentiation into α myofibroblasts. Moreover, TLR4 activation greatly enhanced fibroblast sensitivity to the profibrotic effect of TGF-β. Consistent with these observations, genetic targeting of TLR4, or its endogenous DAMP ligands, or pharmacological disruption of signaling from TLR4 or its co-receptor MD2, ameliorated progressive tissue fibrosis in different disease models [79,80].

Interleukin-33 (IL-33) is a tissue-derived nuclear cytokine from the IL-1 family, including IL-1α, IL-1β, and IL-18 [81]. It is expressed in endothelial cells, epithelial cells, fibroblast-like cells, and myofibroblasts both during both steady-state and inflammation [82,83,84]. As other alarmins, IL-33 is a multitasking molecule. In the inactive state, the full-length IL-33 protein (flIL-33) is associated with chromatin in the cell nuclei and acts as an intranuclear gene regulator [82,85]. The mature form of IL-33 (mIL-33) may be passively released by damaged or necrotic cells upon cell injury, as well as actively secreted by immune cells. It acts as an extracellular cytokine [85,86,87] that alerts a very large number of immune cells expressing the IL-1 receptor-related suppression of tumorigenicity 2 receptor (ST2) (IL-1RL1), a member of the Toll-like receptor (TLR)/IL-1 superfamily [88,89,90,91,92,93,94]. The most important isoforms of ST2 are the membrane-bound receptor (ST2L) and the soluble form (sST2). IL-33 binding to ST2L leads to interaction with IL-1 receptor accessory protein (IL-1RAcP), which clusters their toll/ interleukin-1 receptor (TIR) domains and recruits myeloid differentiation primary response protein 88 (MYD88), the IL-1R associated kinase 4 (IRAK4), IRAK1, and TNF receptor-associated factor 6 (TRAF6) proteins [95,96]. Downstream events, including activation nuclear factor-κB (NF-κB), p38, extracellular signal-regulated kinase (ERK), and c-Jun N-terminal kinase (JNK) [97], stimulate the production of Th2-associated cytokines, IL- 4, IL-5, and IL-13, and support the proliferation and survival of ST2+ cells such as Th2 cells, Treg cells, and ILC2s. Conversely, the IL-33/ST2 signaling pathway is negatively regulated by sST2, which binds to free IL-33 as a decoy receptor, and by single immunoglobulin domain IL-1R-related molecule (SIGIRR), through interaction with the ST2 receptor complex [98]. The main target of IL-33 are cells involved in Th2 immunity and allergic inflammation, such as group 2 innate lymphoid cells (ILC2s), mast cells, Th2 cells, eosinophils, basophils, and dendritic cells. However, the ST2 receptor is also expressed in type-1 immunity cells, like Th1 cells, NK cells, CD8 T cells, neutrophils, macrophages, B cells, and NKT cells. This accounts for the involvement of IL-33 in an extensive range of non-allergic diseases, including fungal, helminth, protozoa, bacterial, and viral infection diseases, cardiovascular diseases, chronic obstructive pulmonary disease (COPD), fibrotic diseases, musculoskeletal diseases, inflammatory bowel diseases, diseases of the central nervous system (Alzheimer), cancer, graft versus host disease (GVHD), obesity, and diabetes. Many studies have also indicated IL-33 as an important factor in the pathogenesis of multiple inflammatory, autoimmune and allergic diseases, such as SLE, rheumatoid arthritis (RA), and inflammatory bowel disease (IBD) [99,100,101,102]. The IL-33/ST2 axis has long been known to play a pivotal role in the development and regulation of immune responses, as well as cell homeostasis, by promoting wound healing and tissue repair. These tissue-protective functions of IL-33 involve activation of tissue-reparative M2 macrophages [103], ILC2s [104], and Tregs [105]. A profibrogenetic role of IL-33 has been demonstrated for pulmonary fibrosis (idiopathic and SSc-related fibrosis), liver fibrosis (cirrhosis, viral hepatitis, primary biliary cirrhosis, and NASH), pancreatic fibrosis, intestine fibrosis (IBD), renal fibrosis, and skin fibrosis. In regard to skin fibrosis, it has been documented that the IL-33/ST2 axis associates with fibroblast proliferation, differentiation of endothelial cells, increased angiogenesis, and ECM deposition [106].

To explore the consequences of the dysregulated IL-33 signaling pathway in the skin, Rankin et al. injected recombinant IL-33 to *IL-1RAcP*-/-, *ST2*-/-, *IL-13*-/-, and WT mice subcutaneously. Interestingly, the development of cutaneous fibrosis was observed in association with ST2 and IL-1RAcP-dependent accumulation of CD3 lymphocytes and eosinophils. IL-33 stimulated bone-marrow-derived eosinophils to secrete IL-13, which might represent a key mediator of IL-33-induced fibrosis [107].

In an initial work on the role of IL-33 in SSc, Manetti et al. studied the IL-33 protein expression, IL33 mRNA, and ST2 expression in skin and visceral organ biopsies from SSc patients (both early and late) and controls. They found that the expression of IL-33 protein in EC and keratinocytes in the skin was markedly decreased in early SSc patients, whereas the mRNA expression was normal or even increased. This was explained as mobilization of IL-33 by EC nuclei in the early disease stage upon activation/damage. By contrast, in patients with late-stage SSc, IL-33 protein was constitutively found in most endothelial cells. The authors then concluded that IL-33 might play an important role as epithelial “alarmin” in tissues exposed to the environment [108]. In subsequent work, the same research group also demonstrated that circulating IL-33 was significantly higher in SSc patients than in controls and early stage SSc than in late-stage SSc. Moreover, IL-33 levels were significantly higher in patients with the “active” than in those with the “late” capillaroscopic pattern. These findings, in association with the reported strong expression of ST2L in endothelial cells, inflammatory/immune cells, and myofibroblasts in early stage SSc, suggested a role of IL-33 in the active derangement of the microcirculation, as well in other pathogenetic mechanisms of SSc, such as immune abnormalities and fibrosis [109].

Yanaba et al. added another piece of information, demonstrating that high serum level of IL-33 positively correlated with skin sclerosis and severity of pulmonary fibrosis. When considering the enhancing activity of IL-33 on Th2 responses, the authors suggested that IL-33 is likely to contribute to the Th2 lymphocyte infiltration and promote Th2 cytokine production, such as IL-4 and IL-13, leading to skin fibrosis in SSc [110]. According to these findings, the expression of IL-33 mRNA is reported to increase in the primary pulmonary fibroblasts from patients with SSc-ILD, as well as in those from patients with idiopathic pulmonary fibrosis (IPF) [111]. These findings were only in part confirmed by a subsequent study of Terras et al. that found a significant association only between the increased levels of Il-33 and peripheral vascular involvement in form as digital ulcers [112]. More interestingly, Vettori et al., when studying the serum levels of factors involved in endothelial, T-cell, and fibroblast interplay in SSc patients, found that IL-33 was significantly higher in early SSc patients, as compared to both controls and SSc patients with a definite disease. The early subset of this study was represented by patients presenting with RP and NVC scleroderma findings and/or SSc marker autoantibodies not meeting 2013 ACR/EULAR criteria for SSc. This newly identified early SSc population represents an ideal setting to investigate endothelial-lymphocyte-fibroblast interplay in the early stages of the disease. In early SSc, there were no differences in the other investigated markers, according to the functional and serological features assessed. Therefore, IL-33 might mediate the very early pathogenic events of SSc, being crucially increased in the early subset. At the same time, the data also suggested that IL-33 gives way to Th2-type pro-fibrotic cytokines, like IL-13, in the fibrotic stages of the disease [113]. More recently, Zhang et al., in a case-control study in the Chinese population, found that serum levels of IL-1β, IL-18, and IL-33 in SSc patients were considerably augmented than those detected in healthy subjects. No correlations of serum IL-1α, IL-1β, IL-18, and IL-33 levels with clinical parameters were found. The authors thus concluded that, although no direct associations between these cytokines and disease manifestations could be demonstrated, they still could be considered as serum markers of the development of SSc [114]

Moreover, sST2 has also been suggested to have a direct role in fibrotic diseases, such as liver fibrosis, possibly representing a suitable biomarker. Wagner et al. found elevated levels of sST2 in late-phase limited cutaneous SSC (lcSSc), as compared to patients with shorter disease duration or with the diffuse subtype of SSc. Since iloprost treatment resulted in lowered sST2 levels, the authors proposed sST2 as a biomarker and possibly a therapeutical target. Recently, MacDonald et al. showed that (i) Treg cells from affected skin of SSc patients produced significant amounts of IL-4 and IL-13, and (ii) Treg cells in the blood of patients with SSc had a considerably higher ratio of skin-homing cells expressing TH2-cell-associated chemokine receptors. The authors also found that IL-33 stimulated the differentiation of skin Treg cells into TH2-like cells and that skin-resident Treg cells expressed the ST2 chain of the IL-33 receptor. Taken together, all of these findings strongly suggest that IL-33 might be an important stimulator of tissue-localized loss of normal Treg cell function. Localized dysfunction of Treg cells might contribute to fibrosis in patients with SSc [115]. A role for IL-33-producing dermal fibroblasts in Th2-like Treg transdifferentiation has been recently suggested by Saigusa et al. They demonstrated that, in the bleomycin-treated Fli1+/− mice model for SSc, Fli1 haploinsufficiency increased proportions of Th2- and Th17-like Tregs [116].

Kurowska-Stolarska et al. showed that IL-33 enhanced the polarization of M2 macrophages in an IL-4Rα- and IL-13-dependent manner. Il-13, in turn, increased macrophage responsiveness to IL-33 by increasing ST2L expression [117]. Activated macrophages are known to produce IL-13, a profibrotic cytokine in pathological fibrosis. In addition, IL-33 also induced the expansion of type 2 innate lymphoid cells (ILC2s), to increase the production of IL-13 [118]. Altogether, these findings strongly suggest that IL-13 represents a key factor for the fibrotic activity of IL-33. IL-13 is a pleiotropic cytokine mainly secreted by activated Th2 cells, and, to date, multiple fibrotic pathways have been demonstrated to be triggered by it. On one hand, IL-13 may stimulate fibroblast activation both in a direct manner and through the production of TGF-β; on the other hand, Il-13 maintains sustained type-2 immune response, as well as increased levels of proinflammatory mediators that play an important role in chronic fibrotic disorders. Lee et al. demonstrated that IL-13 induces tissue fibrosis by selectively stimulating and activating transforming growth factor-beta 1. IL-13 stimulates macrophages to produce TGF-β by several distinct mechanisms, including the production of latent TGF-β and upregulation of matrix metalloproteinases (MMPs), that cleave the LAP-TGF-β complex [119,120], as well as via an IL-13Rα2 signaling pathway. In fact, despite IL-13Rα2 being considered nonfunctional and only acting as a decoy receptor for IL-13, recent studies have shown that signaling is possible through IL-13Rα2 and that, during prolonged inflammation, in the presence of TNF-α, this pathway leads to the production of TGF-β by macrophages and, ultimately, fibrosis in various experimental inflammatory states [121,122]. Interestingly, a significant association between IL-13Rα2 gene polymorphisms and susceptibility to SSc has been found in a French cohort of the Caucasian population [123]. A recent work has demonstrated that a dysregulated production of IL-13 by effector CD8+ T cells is critical for predisposing patients to more severe forms of cutaneous disease. This dysregulation seems to be associated with defects in the molecular control of IL-13 production, such as increased expression of the transcription factor GATA-3. The silencing of GATA-3 with siRNA significantly reduces IL-13 production by CD8+ T cells from patients.

IL-1α is a nuclear alarmin belonging to the IL-1 superfamily of cytokines that comprises 11 cytokines, seven with agonistic functions (IL-1α, IL-1β, IL-18, IL-33, IL-36α, IL-36β, and IL-36γ) and four with antagonistic activities (IL-1Ra, IL-36Ra, IL-37, and IL-38) [124], which amplify and modulate the immune response. Based on both altered expression or gene polymorphisms, about half of the members of this family (IL-1α, IL-1β, IL-18, IL-33, and IL-36α) have been associated with fibrotic diseases, including SSc. Similarly, to most IL-1 family members, IL-1α is expressed as a full-length precursor that needs proteolytic processing to acquire biological activity. The full-length IL-1α is cleaved by cysteine protease calpain, whereas IL-1β and IL-18 precursors are cleaved by the inflammasome [125]. IL-1α bind to IL-1R1 receptor, consisting of extracellular Ig domains and intracellular TIR domains. IL-1R1 induces cell activation recruiting cytoplasmic myeloid differentiation primary response protein 88 (MyD88), IL-1R associated kinase 4 (IRAK4), and tumor necrosis factor receptor-associated factor 6 (TRAF6). This signaling cascade ends up in the activation of NF-κB and mitogen-activated protein kinase (MAPK) [126].

Genome-wide association studies have revealed associations between genes encoding IL-1 family cytokines and SSc. The human IL-1α (IL-1A) gene contains the sodium nitroprusside (SNP) rs1800587, which has been reported to be associated with SSc susceptibility in the Slovak Caucasian, Japanese, and Chinese populations [127]. However, in a very recent meta-analysis, IL-1A rs1800587 polymorphism seems not to be statistically linked to the risk of SSc [128]. Fibroblasts obtained from the lesional skin of SSc patients show an abnormal phenotype in vitro, characterized by altered expression of several receptors, including transforming growth factor-β (TGF-β) receptor and IL-1 receptor type I.

In a pivotal study published in 1994, by Kawaguchi, an increased expression of IL-1α mRNA in cultured SSc fibroblast was reported to be found [129]. Some years later, Higgins et al. demonstrated that lesional fibroblasts from SSc patients also showed increased intracellular IL-1α, as well as intracellular IL-1αR antagonist [130]. Therefore, there may exist intracellular regulatory loops that modulate the expression and activities of the intracellular IL-1 family members, similarly to the case for the secreted IL-1s. Moreover, since SSc fibroblasts expressed elevated basal pre-IL-1α and induction of icIL-1Ra, compared with normal fibroblasts, the authors speculated that these intracellular cytokines might play a role in the pathogenesis of scleroderma.

In another study published in 2004, Kawaguchi et al. added further information about the role of IL-1α in the pathogenesis of SSc. In fibroblasts from SSc patients, the production of pro-collagen and IL-6 were decreased when the expression of IL-1α was inhibited via IL-1α siRNA. As opposite, overexpression of IL-1α through stable transfection in normal fibroblasts induced the differentiation of the SSc fibroblast phenotype [131]. These observations suggested that IL-1α could have a potential role in regulating fibroblast–myofibroblast differentiation, a key event in SSc. At present, evidence of altered IL-1α serum level is lacking. In a very recent paper, Lin et al. reported that SSc patients with high serum IL-1α concentrations were more likely to have digital ulcers [132].

Different mechanisms have been suggested to explain the profibrotic role of IL-1α in SSc. Endogenous IL-1α stimulates the production of IL-6 and PDGF in SSc, as demonstrated by the observation that inhibition of endogenous IL-1α resulted in decreased expression levels of IL-6 and PDGF in SSc fibroblasts [133]. IL-6 is profibrotic in multiple ways, including induction of pro-fibrotic gene expression in vivo, enhancement of TGF-β1 production, and regulation of the TGF-β receptor [134]. TGF-β1 is the main regulator of fibrosis-stimulating epithelial-mesenchymal transition (EMT), fibroblast proliferation, ECM synthesis, and inhibition of collagenase and MMP [135]. PDGF is a potent chemotactic factor for inflammatory cells and TGF-β1 [136]. Alternatively, a profibrotic activity of IL-1α could result from its inhibitory activity on nuclear protein necdin, which has been demonstrated to possess an inhibitory effect on procollagen type I production [137]. Finally, IL-1α and IL-1β were found to promote the viability of cultured fibroblasts and myofibroblasts from patients with SSc [138]. Therefore, IL-1 might contribute to fibroblast–myofibroblast differentiation and the myofibroblast longevity, which are believed to be key events in SSc-consequent skin fibrosis in patients with SSc.

## 3. Expression and Function of Granule-Derived Alarmins α- and β-Defensins and LL-37 in SSc

Defensins are small arginine-rich cationic peptides with antimicrobial activity [139]. Human defensins are cationic peptides of approximately 30 amino acids classified into two subfamilies, namely α- and β-defensins, based on their disulfide bond linkages. As major players at the front line of defense, immunological activities of defensins and their role as alarmins in host defense have been intensively investigated. In humans, among the six known α-defensins, human neutrophil peptides (HNP)-1 to 4 are found in neutrophils, whereas human defensins (HD)-5 and HD-6 are mainly expressed in intestinal Paneth cells and the respiratory and female reproductive tracts. Human β defensins (HBDs) are expressed by epithelial cells in the skin and at mucosal surfaces in contact with the environment. While neutrophils produce the largest amounts of HNPs, these peptides are also found in other immune cells [140]. Accumulating evidence indicates that levels of defensins are often altered not only in response to infection but also in inflammation, angiogenesis [141], or in case of tissue damage, thus suggesting an immune regulatory role in disease pathogenesis [142,143]. Increased levels of HNPs and HBD have been observed in both plasma and BALF from patients with different inflammatory lung diseases [144], in association with increased neutrophil counts [145]. Furthermore, HBDs are chemoattractants for numerous cell types, increase cell proliferation, and accelerate angiogenesis and wound healing. It has been shown that HBD-3 plays a role in skin barrier homeostasis by improving the tight junction (TJ) barrier [146]. Immunomodulatory properties of HBDs are controlled by various pathways, including chemokine receptors, mas-related G-protein-coupled receptor X2 (MrgX2), TLRs, epidermal growth factor receptor (EGFR), and some GPCRs.

The significance of abnormal levels of defensins in SSc is unclear. On one hand, it might simply be the consequence of the cytokine microenvironment of SSc aimed at protecting against infection. On the other hand, it could have pathogenic importance in SSc, as recently suggested by Kuzumi et al., who found significantly reduced levels of HBD-2 in SSc patients, in comparison to healthy controls. HBD-2 levels were significantly lower in patients with diffuse SSc, as compared with limited SSc, and in early stage dcSSc, as compared to mid-stage and late-stage. It is worth noting that SSc patients suffering from telangiectasia, pitting scars, and digital ulcers had HBD-2 levels significantly higher in comparison to patients without symptoms [147]. Increasing HBD-2 levels from early to the late stage of the disease could be related to the shift from a Th2 to a Th 1 profile over the disease course [148] since Th2 cytokine downregulates HBD-2, while Th1 cytokine increases it. HBD-2 might also contribute to the development of vasculopathy in SSc through its angiogenic properties. HBD-2 is expressed in vascular endothelial cells associated with oral squamous cell carcinoma (OSCC) and Kaposi’s sarcoma lesions, but not in that of the normal stroma. Therefore, it has been hypothesized that its expression in vascular endothelial cells located within malignant lesions may play a role in tumor angiogenesis and cancer metastasis [141].

Another finding favoring the involvement of α-defensins in SSc pathogenesis is the correlation found between the increased levels of α-defensins in bronchoalveolar lavage fluid of SSc patients and clinical disease parameters of interstitial lung disease, including ILD biomarkers, pulmonary function tests, the ratio of neutrophils and eosinophils in BALF, tricuspid regurgitation peak gradient (TRPG), and the extent of high-resolution computed tomography (HRCT) findings in the lung [145].

Cathelicidin family peptides are antimicrobial peptides found in many mammalian species, and LL-37 is the only human cathelicidin known so far. LL-37 is generated from the hCAP-18 precursor, which is proteolytically generated by proteinase 3 in neutrophils and kallikrein 5 and 7 in epidermal keratinocytes. LL-37 is produced by many cell types, including neutrophils, macrophages, NK cells, and epithelial cells of the skin, airways, ocular surface, and intestine. Moreover, to exert a wide number of antimicrobial activities against bacteria, viruses, fungi, and parasites, LL-37 carries numerous pro- and anti-inflammatory and chemotactic properties that may contribute to autoimmune disease development (for a comprehensive review, see [149]). Kim et al. demonstrated that LL-37 expression was enhanced in SSc fibroblast, as compared with healthy controls, and that LL-37 inhibited SSc fibroblasts from sodium nitroprusside (SNP)-induced apoptosis through modulation of Bcl-2, as well as activation of caspase-3, COX-2, and the ERK pathway. Therefore, LL-37 could have a role in skin sclerosis by inhibiting the apoptosis of dermal fibroblasts [150]. In a subsequent study, Hizal et al. investigated the circulating levels of LL-37 in SSc patients and its association with clinical, laboratory, and instrumental parameters. The main result of this study was the significant association between the lower levels of circulating LL-37 and the pulmonary involvement defined at HRCT (ground glass, reticular, and honeycombing pattern). No significant association or correlation was found between LL-37 levels and any other clinical, serological, or instrumental features. The authors, therefore, suggested LL-37 as a possible marker for interstitial lung disease [151].

More recently, Takahashi et al. investigated the serum LL-37 levels and the LL-37 expression in lesional skin from both patients and SSc animal models by immunostaining and quantitative RT-PCR. LL-37 expression was increased and correlated with interferon-α expression in systemic sclerosis lesional skin, possibly reflecting LL-37-dependent induction of interferon-α. Similarly, in SSc animal models, bleomycin-treated skin exhibited the expression pattern of CRAMP, a murine homolog of LL-37. Finally, LL-37 levels were significantly higher in SSc patients, in comparison to healthy controls, and positively associated with skin score and the activity of alveolitis and were considerably higher in subjects with digital ulcers compared with those without [152].

## 4. Expression and Function of Cytoplasmic Alarmins HSP-70 and S100 in SSc

The first study reporting involvement of HSP-70 in SSc dates back to 1990, when Deguchi et al. found significantly increased HSP-70 levels in scleroderma fibroblasts by a nuclear run-on transcription assay and Northern blot assay [153]. Subsequently, Ogawa et al. found increased serum levels of Hsp70 in patients with both limited cutaneous and diffuse cutaneous SSc. The HSP70 levels were significantly increased in patients with pulmonary fibrosis or contracture of phalanges, compared with those without these damages. Finally, serum Hsp70 levels associated positively with modified Rodnan total skin thickness score, renal vascular resistance, serum levels of monocyte chemotactic protein-1, CRP, and serum levels of 8-isoprostane [154]. Suggesting for a redox-related role for HSP70 in SSc, Ogawa et al. reported significantly elevated autoantibodies to methionine sulfoxide reductase A (MSRA), one of the antioxidant repair enzymes, in SSc patients with pulmonary fibrosis and cardiac involvement [155]. Anti-MSRA antibodies correlated positively with renal vascular damage and negatively with pulmonary function tests. Interestingly, anti-MSRA antibody levels were linked positively with serum levels of 8-isoprostane and HSP70. More recently, Mišunovà et al. analyzed the expression regulation of two inducible HSP70 genes, namely HSPA1A and HSPA1B, located within the major histocompatibility complex (MHC) in patients with various systemic autoimmune diseases. The author found that the expression of HSPA1A gene alone was significantly upregulated in patients with SSc and other autoimmune conditions, as compared to healthy controls. Only in SSc patients, the increase in HSPA1A was seen to be in association with an increased level of extracellular HSP70 protein [156].

S100 is a family of highly acidic calcium-binding proteins found in many body organs. They have in common the EF-hand motif found on several calcium-binding proteins. The S100 family was found to be reach of subcategories. S100A7 is also known as “psoriasin” and is a small calcium-binding protein of the S100 protein family of 11.4 kDa expressed in psoriatic skin, where it is upregulated. It was later found to be elevated in other inflammatory skin diseases, as well; it was demonstrated as being a potent chemotactic inflammatory protein for neutrophils and CD4 T lymphocytes. Moreover, it was suggested to have an important role in skin-invasive malignant lesions, participating in tumor progression. Successively, some authors suggested an antibacterial action in wounds for this alarmin. Baldini et al. found the psoriasin in the whole saliva of patients with diffuse systemic sclerosis. Thus, it revealed a potential salivary marker ability in patients affected by SSc associated with pulmonary involvement. The authors hypothesized that the link between psoriasin and lung involvement may be represented by the protein chemotactic action for immune cells [157]. More recently, to investigate the potential contribution of psoriasin to the development of SSc, Takahashi et al. analyzed the psoriasin expression in the skin samples and sera derived from SSc patients. The expression of psoriasin was elevated in the epidermis of SSc lesional skin and psoriasin levels were higher in SSc patients, especially in those with late-stage (atrophic phase) diffuse cutaneous disease than in healthy controls. Interestingly, SSc patients with interstitial lung disease, telangiectasia, and pitting scars had significantly augmented levels of serum psoriasin, as compared to those without each of these symptoms. In the subgroup of patients with interstitial lung disease, the elevation of serum psoriasin levels was associated with higher ground-glass opacity scores. Considering the selective expression of psoriasin in the epidermis and its chemoattractant and pro-angiogenic properties, this molecule may contribute possibly through a pro-inflammatory activity on keratinocytes to the development of clinical symptoms in SSc, such as alveolitis, telangiectasia, and pitting scars [158].

A role for S100A8/A9, formerly known as MRp8-14, has been also suggested in the pathogenesis of arthritis and other autoimmune diseases [159]. In a very initial paper, plasma levels of S100A8/A9 were found at elevated levels in the sera of some patients with connective tissue diseases, including systemic sclerosis [160]. More recent papers have confirmed and expanded that observation, reporting an increased expression of S100A8 and S100A9 in the epidermis of SSc patients. Plasma S100A8 levels were increased in diffuse cutaneous SSc (dcSSc) [161] and might serve as a possible biomarker for interstitial lung disease (ILD) [162]. Plasma levels of S100A9 were also increased in SSc patients [161], and since S100A9 induces fibroblast proliferation and fibroblast production of connective tissue growth factor (CTGF/CCN2) through Toll-like receptors, it likely contributes to the development of tissue fibrosis in SSc [163]. Altogether, these observations suggest that epithelial cells play many more roles in SSc than previously believed and that S100 alarmins are key mediators underlying this process.

Alarmins involved in SSc pathogenesis are listed in Table 1, which summarizes their biological activities and the main findings of their roles in SSc.

## 5. Perspectives

Alarmins, as well as their receptors, play fundamental roles in linking innate and adaptive immune responses to initiate host defense against danger signals. A growing body of evidence points to abnormal alarmin responses in many inflammatory and autoimmune diseases, and therefore, targeting alarmins is of great therapeutic potential for fibrotic diseases. However, sterile inflammation is regulated by a complex interaction of a large number of physiologically integrated molecules, and the risk of negative outcomes, such as increased infection, could be elevated altering alarmin signaling. Nevertheless, several different strategies have been shown to successfully inhibiting alarmin-dependent inflammatory processes. Currently, we have limited data on alarmin blockade effects. The sense of interfering with these dangerous signals is to counteract vasculopathy, inflammation, and fibrosis. Interesting results have been obtained in experimental models of different inflammatory diseases with the inhibition of HGMB-1/TLR4-mediated inflammation by P5779 peptide, anti-HMGB1 mAbs, resveratrol, and dexmedetomidine. However, the antibody-based strategy is hindered by the possibility of conformational switches in the tertiary structure of the antibody-recognized region and by the non-cross-reactivity with HMGB2, which may replace HMGB1 as a trigger for inflammation. Another approach to inhibit HMGB-1 consists of the use of molecules which efficiently interact with the main HMGB1-receptor, RAGE, acting as competitive antagonists of HMGB1, such as recombinant box A or S100P-derived RAGE peptide. Other reported molecules affecting HMGB-1 signaling that could be of some interest for SSc are thrombomodulin, haptoglobin, metformin, and glycyrrhizin, a natural triterpene glycoside investigated in several HMGB1-related diseases, proving to inhibit extracellular HMGB1 cytokine activity and protect against ischemia/reperfusion (I/R)-induced injury in animal models. Interestingly, two largely used cholesterol-lowering drugs, namely atorvastatin and simvastatin, significantly diminished the overexpression of HMGB1, RAGE, TLR-4, and NF-kB induced by ischemia [164].

IL-33 is another pro-fibrogenic alarmin potential novel therapeutic target for managing fibrosis in SSc patients. The IL-33 inhibitor (CNTO-7160), which is currently being examined in clinical trials for asthma and atopic dermatitis, may be employed as a new therapy for fibrosis in patients with SSc, since anti-fibrotic effects have been observed on the lung [165,166]. Anti–IL-33 clinical trials are currently ongoing in patients with asthma, food allergy, chronic rhinosinusitis, chronic obstructive and airways disease, and an anti-ST2 is under investigation in patients with chronic obstructive airways disease (for further details, see http://clinicaltrials.gov/). A phase 2a study of a single intravenous 300 mg dose of etokimab, a humanized IgG1/kappa anti–IL-33 monoclonal, in 12 adult patients with moderate-to-severe atopic dermatitis, has recently been published [167].

Inhibition of signaling through the IL-1 family cytokines (e.g., Interleukin 1 receptor antagonist anakinra) has been used in different autoimmune diseases, including RA. However, few studies have explored the clinical benefits in SSc. In an SSc clinical trial rilonacept, an IL-1 receptor fusion protein did not show treatment-related efficacy to placebo, failing to reduce the expression of IL-6, C-reactive protein (CRP), or CCL18 expression [168]. Moreover, S100 proteins have been targeted effectively in preclinical models and in preliminary clinical trials, to treat autoimmune diseases. Targeting the expression and the immunomodulatory effects of S100 proteins is another promising approach for future therapeutic strategies in SSc.

In conclusion, deeper investigations on the role of alarmins in SSc are required to expand our knowledge about the role of innate immune responses in the pathogenesis of this disabling disease, but also to provide the basis for new therapeutic strategies, thereby putting some of the basic findings into practice.

## Figures and Tables

**Table 1:** Alarmins involved in SSc pathogenesis.

Alarmins	Biological Activities (Target Cells)	Serum Levels in SSc	SSc Pathogenesis and References
HMGB-1 (nuclear)	Nuclear/transcriptional regulator Increased expression of genes for proinflammatory factors (neutrophils) Induction of cytokine and chemokine (monocytes, DCs, macrophages, and endothelial cells). Transendothelial migration (monocytes). Proangiogenic; upregulation of adhesion molecules (endothelial cells). Proliferation of naive T lymphocytes; Th1 polarization (T lymphocytes). Procoagulant activity (platelets).	Increased	Promotes pulmonary, renal, and myocardial fibrosis; endothelial damage; coordinate micro thrombosis [57,71,73].
IL-33 (nuclear)	Intranuclear gene regulator Initiation of innate and adaptive type 2 immune responses with the production of IL-4, IL-5, and IL-13. Polarization of M2 macrophages, proliferation of eosinophils, production of IgE, proliferation, and activation of T helper 2 and group 2 innate lymphoid cell (ILC2) (macrophages, ILC2s, mast cells, Th2 cells, eosinophils, basophils, and dendritic cells). Activation and migration of neutrophils to sites of infection (neutrophils).	Increased	Stimulates fibroblast activation; Induces tissue fibrosis (lung, skin); altered microcirculation; immune abnormalities (Tregs transdifferentiation) [108,109,110,111,112,113,118].
IL-1α (nuclear)	IL-1α binds to chromatin and controls homeostatic functions of the cell, like transcription, proliferation, differentiation, or cell death. Physiological manifestations of IL-1 signaling include fever, hypotension, vasodilation, and increased sensitivity to pain. Apical driver of cutaneous inflammation, colon inflammation and cancer, cardiovascular disease, and neural inflammation.	NA	Stimulates production of pro-collagen; regulates fibroblast–myofibroblast differentiation; stimulate the production of IL-6 (profibrotic) and PDGF (chemotactic for inflammatory cells); promotes the viability of fibroblasts [130,131,132,133,134,135,136,137,138].
α- and β- defensins (granule-derived)	Antimicrobial activity. Altered levels of defensins are observed in response to infection, inflammation, angiogenesis or tissue damage. HBDs are chemoattractants for numerous cell types, increase cell proliferation and accelerate angiogenesis and wound healing.	Reduced levels in comparison to healthy controls, but increasing levels from early to late-stage SSc	Possible involvement in vasculopathy [144,145,146,147,148].
LL-37 (granule derived)	Antimicrobial activities against bacteria, viruses, fungi, and parasites; chemotactic; pro- and anti-inflammatory activities and	Increased	Increased in SSc fibroblasts; Inhibits apoptosis of dermal fibroblasts in SSc [150,151,152].
HSP-70 (cytoplasmic)	Stimulates both the innate and adaptive immune systems. The recognition of Hsp70 by immune cells causes initiation of signal transduction which results in the subsequent release of cytokines, including IL-1β, IL-6, IL-12 (macrophages), IFN-γ (T cells), IL-10 (monocytes), and TNF-α (DCs).	Increased	Marker of oxidative stress and disease severity in SSc [153,154,155].
S100 (cytoplasmic)	S100A7 chemotactic inflammatory protein (neutrophils, CD4 T lymphocytes); antibacterial activity in wounds. Dual impact of S100A8/A9 (calgranulin A and B, respectively) on the outcome of inflammatory responses. The secondary release after a preceding stimulus has amplifying effects. Under sterile stress conditions hyporesponsiveness to subsequent inflammatory stimuli.	Increased	Salivary marker in SSc patients with pulmonary involvement [157]. Pro-inflammatory activity on keratinocytes leading to alveolitis, telangiectasia and pitting scars in SSc [158]. Possible biomarker for ILD [162]. S100A9 could contribute to the development of tissue fibrosis in SSc trough fibroblast proliferation and production of connective tissue growth factor [164].
